# Optical Coherence Tomography Based Choroidal Thickness and Its Determinants in Healthy Saudi Population: A Cross-Sectional Study

**DOI:** 10.7759/cureus.34152

**Published:** 2023-01-24

**Authors:** Abdullah Al Marshood, Marcos J Rubio Caso, Abdulrahman AlSaedi, Faisal Almarek, Rajiv B Khandekar, Valmore A Semidey

**Affiliations:** 1 Vitreoretinal Division, King Khaled Eye Specialist Hospital, Riyadh, SAU; 2 Department of Surgery, Unaizah College of Medicine and Medical Sciences (UCM) at Qassim University, Qassim, SAU; 3 Department of Ophthalmology, College of Medicine, Imam Mohammad Ibn Saud Islamic University, Riyadh, SAU; 4 Faculty of Medicine, Department of Ophthalmology, The University of British Columbia, Vancouver, CAN; 5 Research Department, King Khaled Eye Specialist Hospital, Riyadh, SAU

**Keywords:** refractive error, sub-foveal region, optical coherence tomography, enhanced-depth imaging, choroidal thickness, choroid

## Abstract

Purpose

To study choroidal thickness (CT) and its determinants based on optical coherence tomography (OCT) in the healthy adult Saudi population.

Materials and methods

This cross-sectional study was conducted in 2021 at a tertiary eye hospital in Saudi Arabia. The autorefractor-based refractive status (spherical equivalent) of each eye was documented. CT was measured from the enhanced depth OCT images at the fovea to the 1500 µm nasal and temporal to the fovea. CT was defined as the distance from a hyper-reflective line representing retinal pigment epithelium (RPE)-Bruch's membrane to the choroid-scleral junction. The CT was correlated with demographic and other variables.

Results

The study sample included 288 eyes of 144 participants (mean age 31.5±8.3 years; males 94, 65.3%). Emmetropia, myopia, and hypermetropic spherical equivalent were noted in 53 (18.4%), 152 (52.5%), and 83 (28.8%) eyes, respectively. The mean sub-foveal (SFCT), nasal, and temporal CT were 329.4±56.7μm, 302.3±63.5 μm, and 312.8± 56.7μm, respectively. CT varied significantly by location (p* *<0.001). CT was negatively correlated with age (r = -0.177, P* *<0.001). CT in emmetropic and myopic eyes was 319.7±53 μm and 313.1±53 μm, respectively. The difference in CT based on refractive status (p* *= 0.49) or sex was non-significant (p* *= 0.6). Regression analysis suggested that age (p* *<0.001), refractive error (p* *= 0.02), scanning time (p* *<0.001), and scanning location (p* *= 0.006) were significant predictors of CT.

Conclusion

CT measurements of the eyes of healthy Saudis can be used as reference values for studies evaluating CT changes due to various chorioretinal diseases.

## Introduction

The choroid is the middle layer between the sclera and retina in the posterior segment of the eye. This layer helps provide oxygen and nutrients to the retina and removes metabolic waste from the outer part of the retina [1]. The advent of optical coherence tomography (OCT) enabled non-invasive imaging of choroidal and retinal structures. This has been further improved with tools such as enhanced-depth imaging (EDI) OCT and OCT angiography (OCTA) [2,3]. Evaluating healthy eyes in different races helps to establish baseline parameters for assessing choroidal changes in different diseases [4,5]. Choroidal thickness (CT) in healthy eyes, as measured by OCT, is influenced by several factors, including scanning location (sub-foveal, nasal, and temporal to the fovea), age, sex, the refractive status of the eye, and diurnal variability [6-12]. Based on anterior OCT, iris thickness in healthy eyes was studied to evaluate a nomogram for the Saudi population [13]. However, to the best of our knowledge, no study has been published on CT and its determinants in the healthy Saudi population. The study was conducted at a tertiary eye hospital in central Saudi Arabia. This hospital provides a vitreoretinal service to patients referred from across the Kingdom and is a teaching hospital for resident ophthalmologists and subspecialty fellows. The hospital has a digital imaging department to support all units and modern diagnostic equipment managed by experienced and trained technicians. We evaluated a sample of healthy Saudi adults who volunteered to participate in this study to develop a nomogram of CT at the sub-foveal, nasal, and temporal-to-fovea locations using OCT. Further, we studied the correlation between CT and different determinants.

## Materials and methods

The Institutional Research Board approved this study (RP 1850). Written informed consent was obtained from all study participants. This study adhered to the principles of the Declaration of Helsinki. This study was conducted between October 2018 and December 2019. This cross-sectional study enrolled healthy adult Saudis who visited our institution and agreed to participate. Participants aged >20 who declined to participate, with ocular complaints and a history of ocular trauma or surgery, were excluded. To calculate sample size, we used six strata: males aged <30, females aged <30, males aged 31-45, females aged 31-45, males aged >45, and females aged >45 with a representative population of 5000, 5000 and 500 for the three age groups, respectively. To achieve 95% CI, an absolute precision of 0.1 and a population in a stratum of 5000, 5000, and 500 in three age groups, respectively, and 50 eyes were required in each stratum. Therefore, the total sample size for the proposed study was 300 eyes, with 50 in each age group. Three ophthalmologists were the field investigators. The Digital Imaging, Optometry, and Research Department staff assisted the investigators. Patient demographics, including age and sex, were collected. The refraction of each eye was measured using an autorefractor (OPD-Scan III, NIDEK Co. Ltd, Gamagori, Japan). 

The spherical and cylindrical refractive error (RE) values of each eye were used to estimate the spherical equivalent (SE) using the formula: spherical RE (in diopters) + [cylinder RE (in diopters)/2]. SE < ±0.1 D was considered emmetropic, < -0.1 D was considered myopic, and > +0.1 D was considered hyperopic. Myopia was further classified as high myopia (SE: >-6.0 D), moderate myopia (SE: -3.0 to -5.9 D), and mild myopia (SE: -0.1 to -2.9 D) [14]. OCT scans were obtained using Spectral Domain-OCT (SD-OCT) (Revo Nx, Optopol, CA, USA). A 12 mm horizontal foveal line scan was obtained for each eye to measure CT at three locations: sub-foveal (SFCT), 1500 μm nasal, and 1500 μm temporal to the fovea. CT was measured manually from a hyper-reflective line representing RPE-Bruch's membrane to the choroid-scleral junction. Measurements were taken by three ophthalmology consultants and then averaged. The data were entered into a Microsoft Access® spreadsheet (Microsoft Corp., Redmond, WA, USA). After a consistency check and removal of duplicate entries, the data were transferred to SPSS 25 (IBM, Armonk, NY, USA). Univariate analysis was performed using a parametric method. Qualitative variables are presented as numbers and percentages. Normally distributed quantitative variables are presented as mean and SD. To compare CT in subgroups, we used difference of means, 95% CI, and two-sided p-values. The correlation coefficient and two-sided p-values were used to investigate correlations between CT and qualitative variables. The variables that were significantly correlated with CT in the univariate analysis were included in the linear regression model. Using a step-out method, predictors of CT were evaluated. A p-value <0.05 was considered statistically significant.

## Results

**Figure 1:**
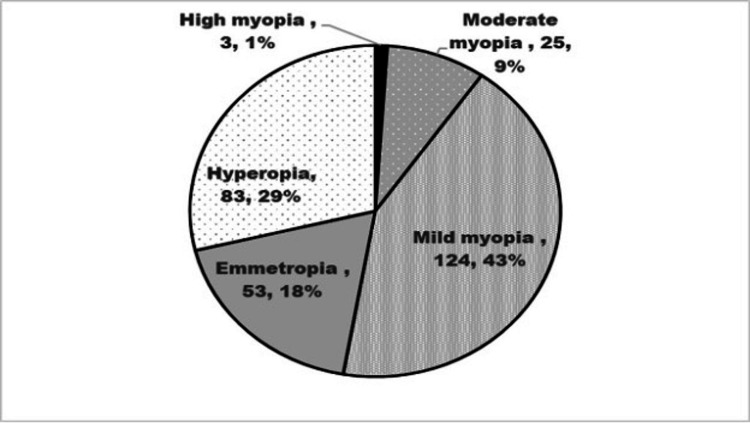
Refractive status in 288 eyes of 144 healthy Saudi adults participating in the study to evaluate choroidal thickness using optical coherence tomography.

The study included 288 eyes of 144 adult Saudis. The mean age was 31.5±8.3 years; 50 (35.7%) were women, and 94 (65.3%) were men. Figure 1 presents the refractive status of 288 eyes. Eyes with high and moderate myopia constituted 10% of the study sample. 

**Figure 2:**
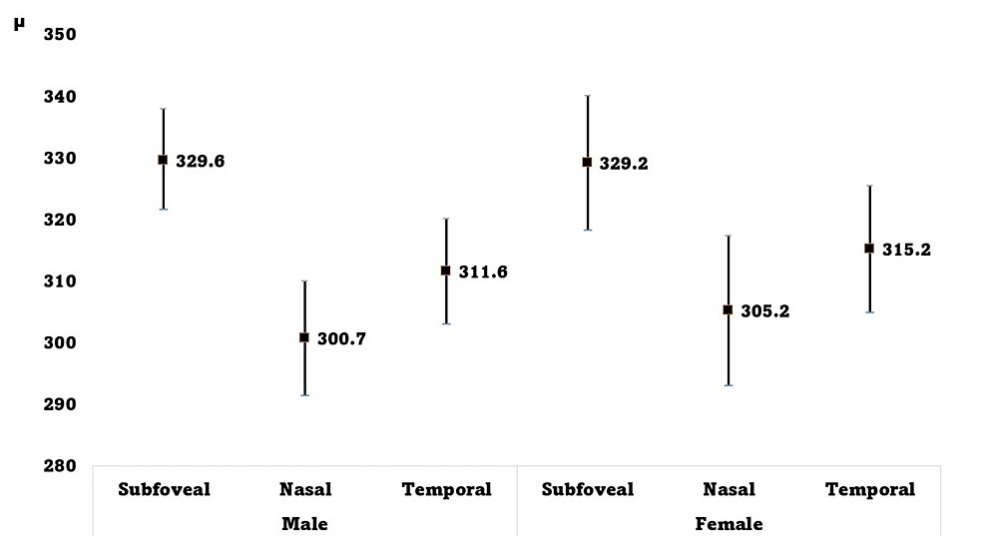
Choroidal thickness (CT) measured by optical coherence tomography at three locations of the retina in 288 eyes of 144 healthy adult male and female Saudi participants. The X-axis denotes sub-foveal, nasal, and temporal horizontal sites 1500 µm away from the fovea in male and female participants. Y axis denotes CT (µm). The square inside the vertical bars denotes the mean value of CT. The end of the vertical bars denotes the upper and lower values of the 95% CI for CT.

The mean CT measured at three retinal locations was 314.8±60.0 µm (95% CI: 307.9-321.7 µm). Table 1 presents the CT at three retinal locations. Sub-foveal CT was significantly higher than nasal and temporal CT (p <0.001). The CT values were similar between sexes at each location (sub-foveal: p = 0.96, nasal: p = 0.6, and temporal: p = 0.6) (Figure 2). 

**Table 1:** Choroidal thickness as measured by optical coherence tomography (OCT) at three locations on the retina in 288 eyes.

	Choroidal thickness (CT)		
Location	Mean (µm)	SD (µm)	95% CI
Sub foveal	329.4	56.7	322.8; 336.0
1500 µm nasal to the fovea	302.3	63.5	294.9; 309.7
1500 µm temporal to the fovea	312.8	56.7	306.2; 319.4

CT at 1500 µm nasal to the fovea was significantly thinner in eyes with high and moderate myopia than in eyes with mild myopia, emmetropia, and hyperopia (Table 2). This difference was statistically non-significant at the sub-foveal (p = 0.1) and temporal (p = 0.3) locations. CT of the right eye compared with the left eye of the same adult was not significantly different (matched pair p = 0.53).

**Table 2:** Choroidal thickness by type of refractive error. P<0.05 is statistically significant.

Sub foveal	Number	Mean	SD	Validation
Mild myopia	124	332.7	56.0	P = 0.1
Moderate myopia	25	301.5	51.7	
High myopia	3	364.0	24.2	
Emmetropia	53	333.0	51.1	
Hyperopia	83	329.5	61.7	
1500 µ nasal to fovea				
Mild myopia	124	308.6	62.5	P = 0.005
Moderate myopia	25	259.8	57.9	
High myopia	3	251.7	57.5	
Emmetropia	53	308.2	54.3	
Hyperopia	83	303..6	67.8	
1500 µ temporal to fovea				
Mild myopia	124	316.1	58.1	P = 0.3
Moderate myopia	25	291.0	54.7	
High myopia	3	303.7	20.4	
Emmetropia	53	318.0	54.2	
Hyperopia	83	311.4	57.0	

SFCT, nasal CT, and temporal CT were negatively correlated with participant age (p = 0.001, 0.001, and 0.009, respectively). SFCT, nasal CT, and temporal CT showed a correlation with the time of day when they were measured (Pearson's correlation r = -0.212, -0.141, and -0.169, respectively; p = 0.0003, 0.017, and 0.004, respectively). The CT measured before noon was significantly greater than the CT measured after noon (mean difference 12.2 μm; 95% CI: 4.1-20.3; p = 0.003). Linear regression analysis was carried out to study the predictors of CT (Table 3). Age (p <0.001), refractive error (p = 0.02), the timing of the scan (p <0.001), and retinal location of the scan (p = 0.006) were statistically significant predictors of CT. 

**Table 3:** Predictors of the choroidal thickness measured by optical coherence tomography in the eyes of healthy Saudi adults. P<0.05is statistically significant. F = 17.16, P <0.001

	Unstandardized B	Standard error	t	Standardized B	95% CI	P-value
Age	-1.30	0.24	-5.32	-0.18	-1.8; -0.8	<0.001
Time in day for scan	-2.36	0.50	-4.73	-0.16	-3.3; -1.4	<0.001
Refractive error (SE)	-0.92	0.40	-2.32	-0.08	-1.7; -0.1	0.021
Site on retina of scan	-8.3	2.41	-3.44	-0.11	-13.0; -3.6	0.0006
Constant	410.4	11.7	35.0		387.4; 433.4	<0.001

## Discussion

In this study, we measured CT using OCT in the eyes of healthy Saudi adults. CT appeared to decrease with age. Moderately and highly myopic eyes had thinner choroids than emmetropic eyes, mildly myopic eyes, and hyperopic eyes at 1500 µm nasal to the fovea. The sub-foveal choroid was thicker than the nasal and temporal choroids (1500 µm distant from the fovea). OCT measurements performed early in the day yielded higher values than measurements performed in the afternoon or later, suggesting diurnal variations. We found no differences in CT between the sexes. To the best of our knowledge, this is the first study of a CT nomogram and factors affecting CT in a healthy adult Saudi population. Indices based on these normal values and morphological and vascular impacts on the choroid in Saudi/Arab populations can be compared to understand the pathophysiology of vitreoretinal diseases in this patient population. As we carefully excluded patients with vitreoretinal pathology or past ocular surgeries, the findings of the present study can be representative of a healthy adult population. We found that the sub-foveal CT was 329 µm. In healthy adults in Western and Far East Asian countries, central choroidal thickness ranges from 272 to 448 μm [15-18]. The values noted in the present study were greater than those reported by Margolis R et al. [19], who evaluated 30 American patients (287 ± 76 µm). In our study, CT at 1500 µm nasal and temporal to the fovea location was thinner than at the sub-foveal location. A US study reported a mean CT of 147±57 µm, 3 mm nasal to the fovea [5]. Several factors, such as software, OCT light source, ethnicity, age, refractive error, or axial length of the participant's eye, were the reasons for variations in CT in different studies [15]. Therefore, one should be careful while comparing our results with the nomogram of other studies. 

In our study, age was negatively correlated with CT. This outcome concurs with studies by Margolis R et al. [19] and Ooto S et al. [20]. Li XQ et al. [11] that used choroidal volume instead of thickness and noted a negative correlation between age and CT. CT was divided into a vascular and total area, which decreased with age [7]. Anatomically and functionally, the choroid seems to weaken with age and changes due to reduced blood supply to the outer retinal layers and pigment epithelium, posing a disease risk. We found no significant difference in CT by sex. This agrees with the observations of Ruiz-Medrano J et al. [7]. However, studies from Denmark and Japan reported greater choroidal volume in males compared with females [11,20]. Interestingly, in healthy Saudi adults, the iris is thicker in males than in females [13]. Differences in CT by sex in the Arab race in our study and studies on Asian and European populations need further confirmation with a larger sample size. We found that CT at 1500 µm nasal to the fovea in eyes with high and moderate myopia was thinner compared with emmetropic and hyperopic eyes. This was also noted in a study from Egypt in which researchers compared the CT of myopic eyes with that of emmetropic eyes [12]. Sub-foveal choroidal thinning in myopia may be linked with the axial length of the globe that causes chorioretinal stretching, resulting in myopic degeneration. In the current study, the study participants who were scanned before noon had thicker choroids than those who were scanned after noon. There was a negative correlation between CT and the timing of OCT scanning, confirming the diurnal variation of CT. Similarly, diurnal variation was noted in the choroidal vascular index in sub-foveal scans [8]. Therefore, clinicians should consider the timing of OCT scanning when interpreting CT to understand physiological and pathological changes in the eye.

CT measurements found in adult Saudis can be useful for OCT manufacturers to incorporate a normative database for the Arab population. There is a significant need for normative OCT databases from various ethnicities, and OCT manufacturers have been slow to respond. Ophthalmologists and those interpreting OCT scans should consider the influence of demographic variables, refractive status, and diurnal variation on CT. Further studies are recommended to correlate anatomical findings of the choroid in the present study with vascular indices in Saudi adults using OCTA. Blood pressure affects the vascular component of the choroid. A study is recommended to investigate the correlation between blood pressure and CT [2,3]. Due to a cross-sectional design, this study had some limitations. The relationship between the outcome and determinants in relation to time cannot be established. Therefore, no causal association should be inferred. However, the demographic factors were established before CT measurements; therefore, they can be termed risk factors. The adult sample examined in the present study was not randomly selected and did not represent the entire healthy population. Therefore, we suggest a judicious interpretation of the results. The axial length influences refractive error, especially in high myopia. We did not collect this information due to logistical problems. Anatomical parameters are usually described using the histology of cadaveric or enucleated eyes. OCT and OCTA scans now enable healthcare providers to review dynamic changes in CT, monitor the progression of diseases affecting the choroid, and study the impact of therapeutic interventions. However, before interpreting CT, the factors identified in the present study that affect CT should be reviewed.

## Conclusions

Using OCT, we studied anatomical indices of the choroids of healthy Saudi adults. The sub-foveal CT in the Saudi population was thinner than in the Oriental and Far East Asian populations. Increasing age, high myopia, location on the retina, and diurnal variation influenced the CT. Establishing baseline CT measurements in our healthy population is a helpful tool in monitoring the presence, progression or response to treatment of a wide variety of chorioretinal disease.
